# Structural variations in papaya genomes

**DOI:** 10.1186/s12864-021-07665-4

**Published:** 2021-05-10

**Authors:** Zhenyang Liao, Xunxiao Zhang, Shengcheng Zhang, Zhicong Lin, Xingtan Zhang, Ray Ming

**Affiliations:** 1 College of Life Science, Center for Genomics and Biotechnology, Fujian Provincial Key Laboratory of Haixia Applied Plant Systems Biology, Fujian Agriculture and Forestry University, Fuzhou, 350002 Fujian China; 2 Department of Plant Biology, University of Illinois at Urbana-Champaign, Urbana, IL 61801 USA

**Keywords:** *Carica papaya*, Structural variation, WGCNA, Domestication

## Abstract

**Background:**

Structural variations (SVs) are a type of mutations that have not been widely detected in plant genomes and studies in animals have shown their role in the process of domestication. An in-depth study of SVs will help us to further understand the impact of SVs on the phenotype and environmental adaptability during papaya domestication and provide genomic resources for the development of molecular markers.

**Results:**

We detected a total of 8083 SVs, including 5260 deletions, 552 tandem duplications and 2271 insertions with deletion being the predominant, indicating the universality of deletion in the evolution of papaya genome. The distribution of these SVs is non-random in each chromosome. A total of 1794 genes overlaps with SV, of which 1350 genes are expressed in at least one tissue. The weighted correlation network analysis (WGCNA) of these expressed genes reveals co-expression relationship between SVs-genes and different tissues, and functional enrichment analysis shows their role in biological growth and environmental responses. We also identified some domesticated SVs genes related to environmental adaptability, sexual reproduction, and important agronomic traits during the domestication of papaya. Analysis of artificially selected copy number variant genes (CNV-genes) also revealed genes associated with plant growth and environmental stress.

**Conclusions:**

SVs played an indispensable role in the process of papaya domestication, especially in the reproduction traits of hermaphrodite plants. The detection of genome-wide SVs and CNV-genes between cultivated gynodioecious populations and wild dioecious populations provides a reference for further understanding of the evolution process from male to hermaphrodite in papaya.

**Supplementary Information:**

The online version contains supplementary material available at 10.1186/s12864-021-07665-4.

## Introduction

Structural variations are generally considered as complex genomic DNA mutations that can affect genome size in species. SV include insertions, deletions, inversions, transposable elements and copy number variations. Compared to single nucleotide polymorphisms (SNPs) and short insertions and deletions (Indels), SVs usually consist of relatively long DNA changes.

Structural variations are ubiquitous in the plant genome and play important roles in several important biological processes [1]. SVs in genome can directly affect gene expression and traits of biological individuals. Corn weight directly affects yield and is one of the key selective traits in the process of domestication and improvement of corn. 521 maize samples were re-sequenced, and B73 and Mo17 were used as reference genomes to construct a structural variation map of corn [2]. It was found that structural variations were more likely to cause changes of gene expression than SNPs. A locus (qHKW1) that controls both grain shape and weight was located on chromosome 1 of maize, and *ZmBAM1d* gene was found to positively regulate maize grain weight. Comparing the *ZmBAM1d* gene region of tropical small grain varieties and B73, a structural variation directly related to grain weight phenotype was found [2]. Structural variation plays an important role in the domestication and evolution of plants. During grape genome evolution, structural variation accumulates in asexually reproduced grape lineages through recessive heterozygotes, and strong purifying selection acts against these SVs. Structural variations distinguish outlier regions of genetic divergence between wild grapes and cultivars, suggesting that they might play an important role in domestication. These outliers regions include a sex-determination region and grape-berry coloring sites [3].

Copy number variations (CNVs) are defined as structural variations resulting from gain or loss of a DNA fragment with a length of larger than 1 kb. It was originally found in the human genome and associated with multiple diseases, and is often used in the prevention and clinical diagnosis of human diseases. CNVs were prevalent in plant genomes, such as Arabidopsis [4, 5], rice [6], corn [7], soybean [8], potatoes [9], and cucumber [10]. CNVs can alter gene dosage and expression, and cause phenotypic traits mutations such as plant height, flowering time, and seed dormancy. The copy number variation of different wheat cultivars Ppd-D1 and Vm-A1 was detected, and the gene expression was found to be associated with copy number variation. Gene expression is elevated with the increase of gene *Ppd-B1* copy number in wheat varieties with different photoperiod sensitivity, so wheat showed insensitivity to photoperiod response. The vernalization requirement is extended with the increase of *Vrn-A1*gene copy number among wheat cultivars with different vernalization requirement, and those with more copies of *Vrn-A1* gene have lower gene expression rate than low-copy cultivars, underlying the important roles of vernalization in promoting flowering in wheat [11]. The wheat dwarf stem gene *Rht-D1c* (*Rht10*) originated from *Rht-D1b* through a tandem duplication event. The increased copy number of *Rht-D1* effectively reduces plant height, and its dwarfing ability is more than three times that of a single copy [12]. CNVs are also related to plant biotic and abiotic stress adaptations, defense responses, adaptive evolution, species formation and heterosis. Genome-wide structural variation studies were performed on 20 representative cultivars of Asian rice (2 indica rice groups, *Indica*, *aus* and 4 japonica rice groups, *rayada, arromatic, tropical japonica*, and *temperate japonica*) and 2886 CNVs were found. Functional annotation analysis of genes located in the CNV region or overlapping with CNV found that they were closely related to specific biological functions such as cell death, protein phosphorylation, defense response, and resistance [13]. Re-sequencing 302 soybean varieties, including 62 wild soybeans, 130 landraces and 110 improved cultivars, found that 162 CNVs were potentially involved in domestication and improvement, and might contribute to important agronomic traits, such as hilum color and soybean height [8]. CNVs can protect soybean from the most destructive pathogen, soybean cyst nematode (SCN). Overexpression of a single copy gene *rhg1-b* in soybean roots is not efficient to prevent SCN. Simultaneous overexpression of several copies of *rhg1-b* might enhance soybean resistance to nematodes [14].

Papaya (*Carica papaya* L.) is a major tropical fruit, which belongs to the small family Caricaceae. The papaya plants grow rapidly and bloom in 3–4 months, and can harvest fruits in about 9 months. It is a rare plant species that continue to flower and harvest fruits throughout the year once it starts to bloom. Papaya is diploid with 9 pairs of chromosomes and its genome was sequenced in 2008 [15]. Papaya is a rare trioecious species, containing female, male and hermaphrodite plants. The sex determination is controlled by a pair of recently evolved sex chromosomes, with XX representing female, XY male, and XY^h^ hermaphrodite plants [16, 17]. Male and hermaphrodite papaya shared a highly similar non-recombining region in chromosome 1, MSY and HSY, respectively, with 99.6% sequence identity [18]. The MSY and HSY regions have a length of about 8.1 Mb and the corresponding X region has a length of 3.5 Mb [19]. Large-scale genome resequencing revealed that the hermaphrodite plants were domesticated from male plants about 4000 years ago [20]. All wild papaya populations are dioecious, including half male plants and half female plants, however cultivated papayas are mainly gynodioecious, including two-thirds of hermaphrodites and one-third of females.

There are obvious differences of morphology and reproduction between the three sex types of plants. Papaya female flowers have an ovary and grows on the stem of plant, that is, in the axils of petioles. The ovary must first receive the pollen of another plant (male or hermaphrodite), as no sign of stamens are found in female flowers, and then it can be fertilized and produce fruit that contains seeds. Hermaphrodite flowers have mature pistils and stamens. They are reproduced in the leaf axils like female flowers, with short peduncles and few secondary flowers. Hermaphrodite plants are the preferred type for papaya commercial cultivation and used for reliable fruit production because they can self-pollinate and do not require male papaya plants nearby. However, high temperatures and water stress will lead to a sex change of hermaphrodite to male. Male flowers are clearly different from other types of flowers because they have branched, drooping, and long peduncles, with multi-inflorescences. They contain stamens with a lot of pollen and aborted pistils inside the flowers that cannot develop into fruit [21, 22].

The genetically modified papaya cultivar ‘SunUp’ is resistant to papaya ring spot virus and is produced from its progenitor cultivar ‘Sunset’ through genetic modification technology [23]. There were 1200 large structural variants that were detected through a genome-wide comparative analysis between “SunUp” and “Sunset”, but no gene was found to be related with plant growth and development [24]. Although SVs have been studied at the level of a single genome, it has not been studied in the papaya population. Here, we identified SVs from 25 wild male samples and 42 cultivated hermaphrodite samples. Then genetic differentiation analysis found that some selected genes were related to different traits of males and hermaphrodites. Finally, we independently examined genes with CNVs by comparing wild populations with different cultivated subpopulations. Our research reveals the role of structural variation in the domestication process from male papaya to hermaphrodite papaya as well as in the sexual reproduction and environmental adaptability evolution in papaya populations.

## Results

### Detection of structural variations using whole genome re-sequencing data

Sixty-seven re-sequencing individuals were used to detect structural variations and the reads were mapped to our improved papaya reference genome (unpublished data), which was generated by incorporating 100 x PacBio long reads and 100 x Hi-C (high-throughput chromatin confirmation) reads. The re-sequencing data has an average coverage of 27.12x, with the minimum 8.82x for sample HCAR309 and maximum coverage 51.88x for HongRi3 (Supplemental Table S1).

A comprehensive evaluation of SV detection tools revealed that the Manta program outperformed other related algorithms in NGS (Next-Genomic Sequencing) reads and thus was included in our analysis [25]. A total of 11,201 structural variants were detected. To improve the accuracy of SVs, we used a 500-bp sliding window to filter SVs and finally selected a length of 200 kb as the threshold. Based on the quality control, 361 partially-assembled insertions were removed from further analysis. Finally, 8083 SVs were retained, including 5260 deletions, 552 tandem duplications and 2271 insertions. The average sizes of these SVs are 3408 bp for deletions, 18,486 bp for tandem duplications and 121 bp for insertions, respectively (Supplemental Table S2).

**Fig. 1:**
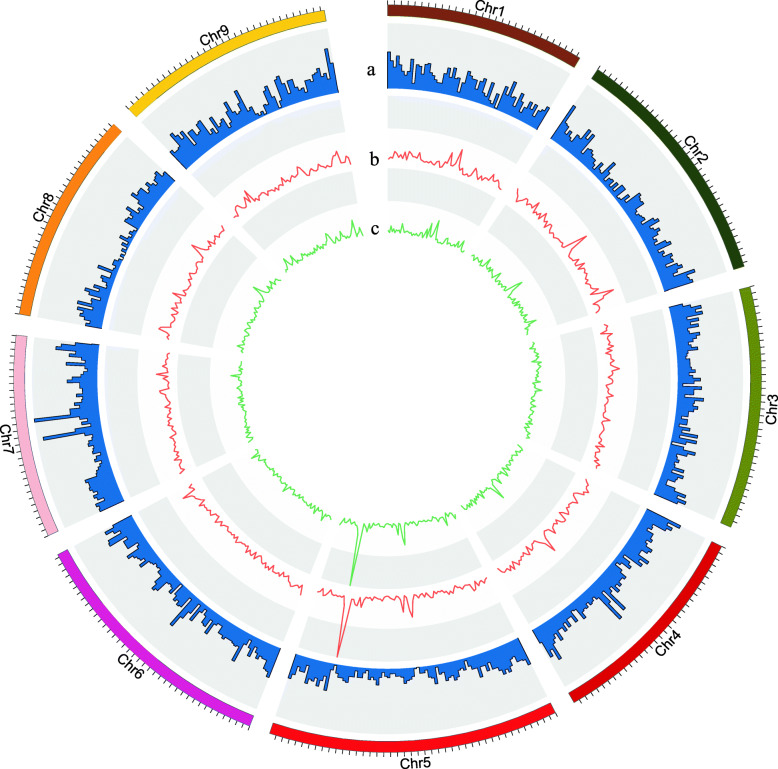
Circle diagram of the distribution of structural variation and copy number variation in the papaya genome. **a**. the distribution of structural variation on different chromosomes. **b**. the distribution of copy number variation in the whole genome of the common cultivated population. **c**. the distribution of copy number variation in the whole genome of the solo cultivated population

We further investigated the distribution of SVs based on 500-kb sliding windows (Fig. 1), showing an uneven distribution pattern across the nine chromosomes. Most of these SVs were mainly concentrated on both ends of chromosomes, such as chr2, chr3, chr4, chr6, chr5, chr8 and chr9. In contrast, a large proportion of SVs were enriched in the middle in chr7.

### Functional impact of SV-overlapped genes

**Fig. 2:**
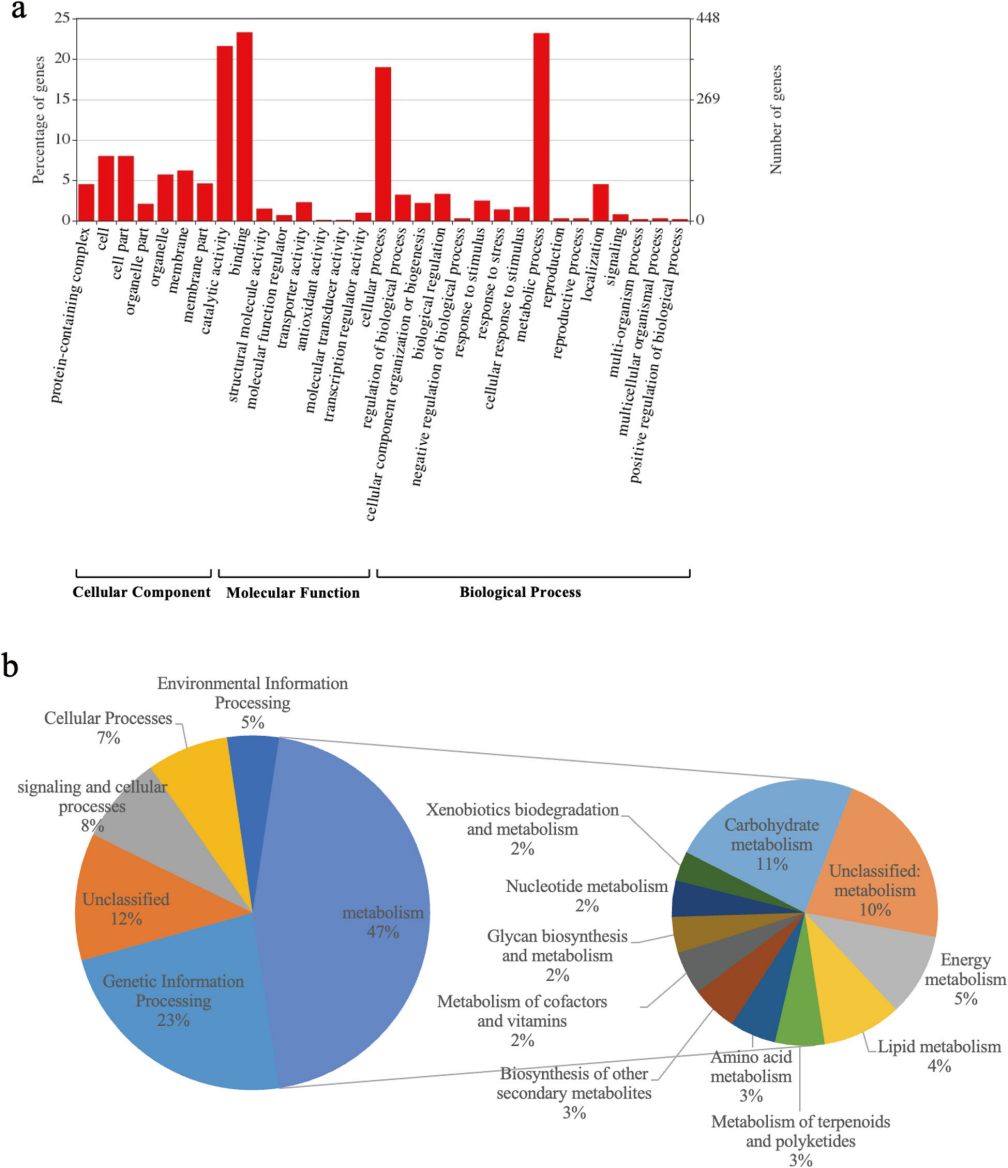
Annotation of structural variations genes function. **a**. The functional annotation of SVs-genes in molecular function (MF), biological process (BP) and cellular component (CC) gene ontology three categories. **b**. The distribution of SV-genes KEGG function annotation pathway

To assess the possible functional impact of these structural variations, we further investigated genes overlapping with these variations. We used in-house scripts and found that a total of 1794 genes overlapped with SVs. The functional implication of the SV-affected genes were investigated based on Gene Ontology (GO) annotation analysis, and Kyoto Encyclopedia of Genes and Genomes (KEGG) annotation analysis. The 1794 genes were categorized into all three primary gene GO categories, molecular function (MF), biological process (BP) and cellular component (CC) (Fig. 2a). Some classes related to environmental response were found in biological process, such as response to stimulus (GO:0050896), response to stress (GO:0006950), and so on. According to the results of KEGG annotation analysis, 47% of genes were related to metabolic pathways, including carbohydrate metabolism (11%), unclassified metabolism (10%), Energy metabolism (5%), and lipid metabolism (4%) [26] (Fig. 2b).

To investigate the impact of SVs on genes, we classified 557 SVs located in coding regions, 1326 locating in introns and 6200 in intergenic regions. Those SVs appearing in the coding regions were identified as large-effect SVs, which overlapped with 761 protein-coding genes. Go enrichment analysis revealed that these genes were mapped in eight significant GO sub-categories (*P* < 0.05). Among them, most genes were enriched in DNA integration and protein dimerization activity (Supplemental Fig. S1). Other activities included terpene synthase activity, serine-type endopeptidase inhibitor activity and lyase activity. We also found that these genes were enriched in response to wounding, FAD binding and ADP binding. In addition, KEGG enrichment analysis revealed that these genes were mainly involved in three meaningful pathways, glutathione S-transferase, multidrug resistance protein, MATE family, and laccase, respectively (Supplemental Fig. S2).

**Fig. 3:**
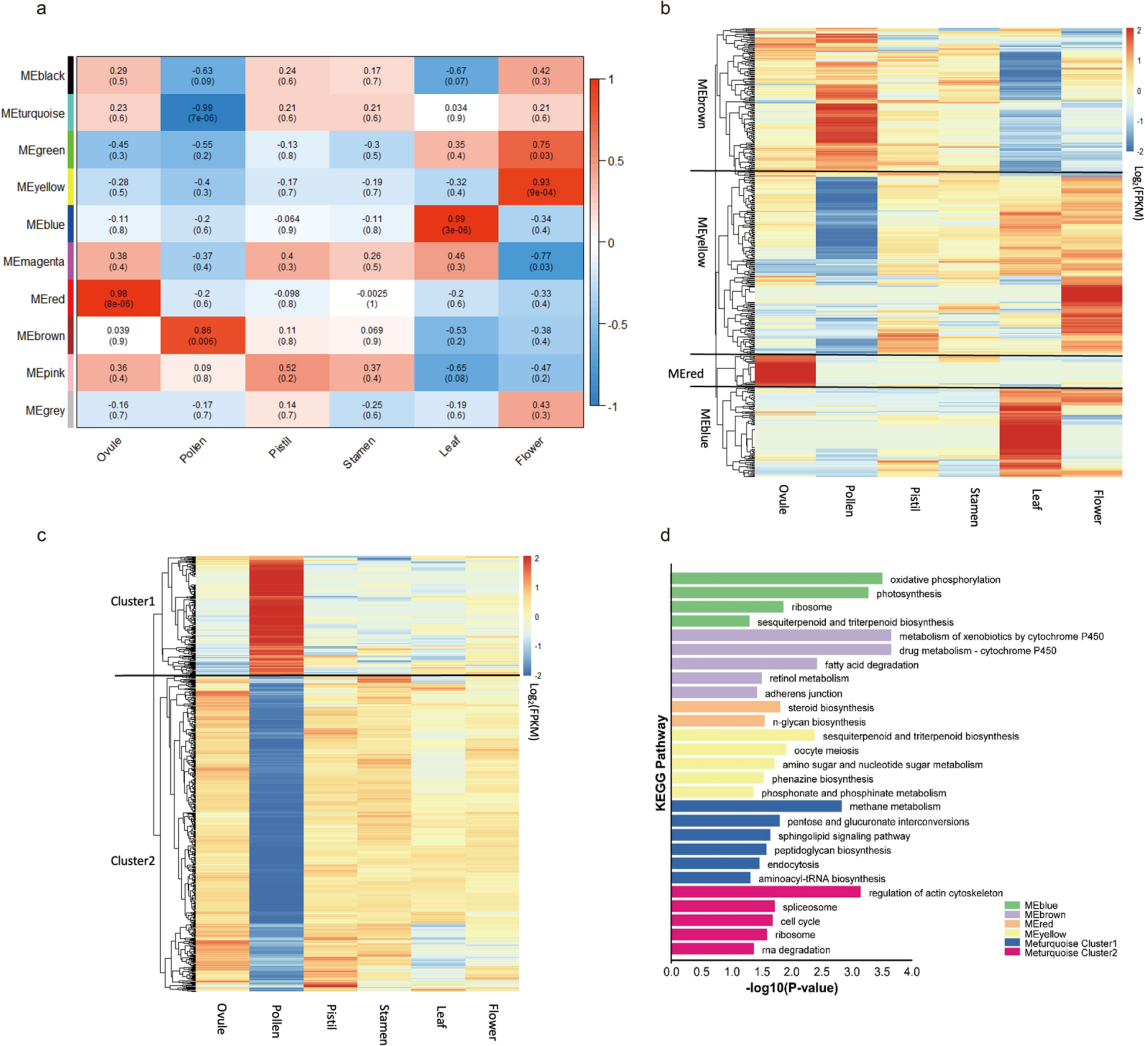
weighted correlation network analysis result graph. **a**. Correlation diagram of modules and traits in WGCNA analysis. **b**. Heatmap of the expression of genes with different positive regulatory modules (MEblue, MEbrown, MEred, and MEyellow) in different tissues. **c**. The heatmap of negative regulation module (MEturquoise) genes expression in different tissues. **d**. KEGG enrichment analysis of different modular genes

To reveal the expression of these 1794 genes in papaya, transcripts data from 6 different tissues were analyzed, ovule, pollen, pistil, stamen, leaf and flower, respectively. Among them, the expression of 444 genes was 0 in all tissues, and the remaining 1350 genes were subject to WGCNA analysis to reveal the co-expression relationship between genes and different tissues. The WGCNA takes advantage of the correlation between genes, group genes into modules using network topology, and combines modules with module similarity greater than 0.8 to get the final gene module classification (Supplemental Fig. S3). A total of 10 modules were identified, different tissues were regarded as trait, the most optimal-related modules of feature vector genes and phenotypes were screened, and the heat map of module-trait relationships was drawn. Four modules with extremely strong positive correlation with trait were identified, and the correlation coefficient (CC) between MEred module and ovule reaches 0.98 (*P* value = 8 × 10^− 6^), MEbrown and pollen (CC = 0.86, *P* value = 0.006), MEblue and leaf (CC = 0.99, *P* value = 3 × 10^− 6^) and MEyellow and flower (CC = 0.93, *P* value = 9 × 10^− 4^), respectively (Fig. 3a). Transcriptional data analysis revealed that the positively correlated module genes were highly expressed in the corresponding tissues (trait). Forty-one MEred module genes were highly expressed in ovule; 106 MEbrown module genes had the highest expression in pollen tissues; 109 leaf highly expressed genes were clustered in MEblue module; and 251 highly expressed genes in flowers were identified to MEyellow module (Fig. 3b). The KEGG enrichment analysis of module genes revealed that they were mainly enriched in some pathways related to biosynthesis, physiological and biochemical reactions and metabolism (Fig. 3d), such as sesquiterpenoid and triterpenoid biosynthesis, steroid biosynthesis, oxidative phosphorylation, photosynthesis, amino sugar and nucleotide sugar metabolism, and fatty acid degradation. In addition, an extremely strong negative correlation between MEturquoise module and pollen was found and the correlation coefficient reached to 0.99. The genes of the MEturquoise module were clustered into two categories (cluster 1 and 2) based on the gene expression pattern in pollen. The cluster 1 included genes that were highly expressed in pollen, and in contrast, genes with extremely low expression in pollen were classified into cluster 2 (Fig. 3c). Functional enrichment analysis showed that the MEturquoise module genes were also involved in biosynthesis, metabolism and biological regulation [26] (Fig. 3d).

### Phylogenetic tree and domesticated structural variant genes

**Fig. 4:**
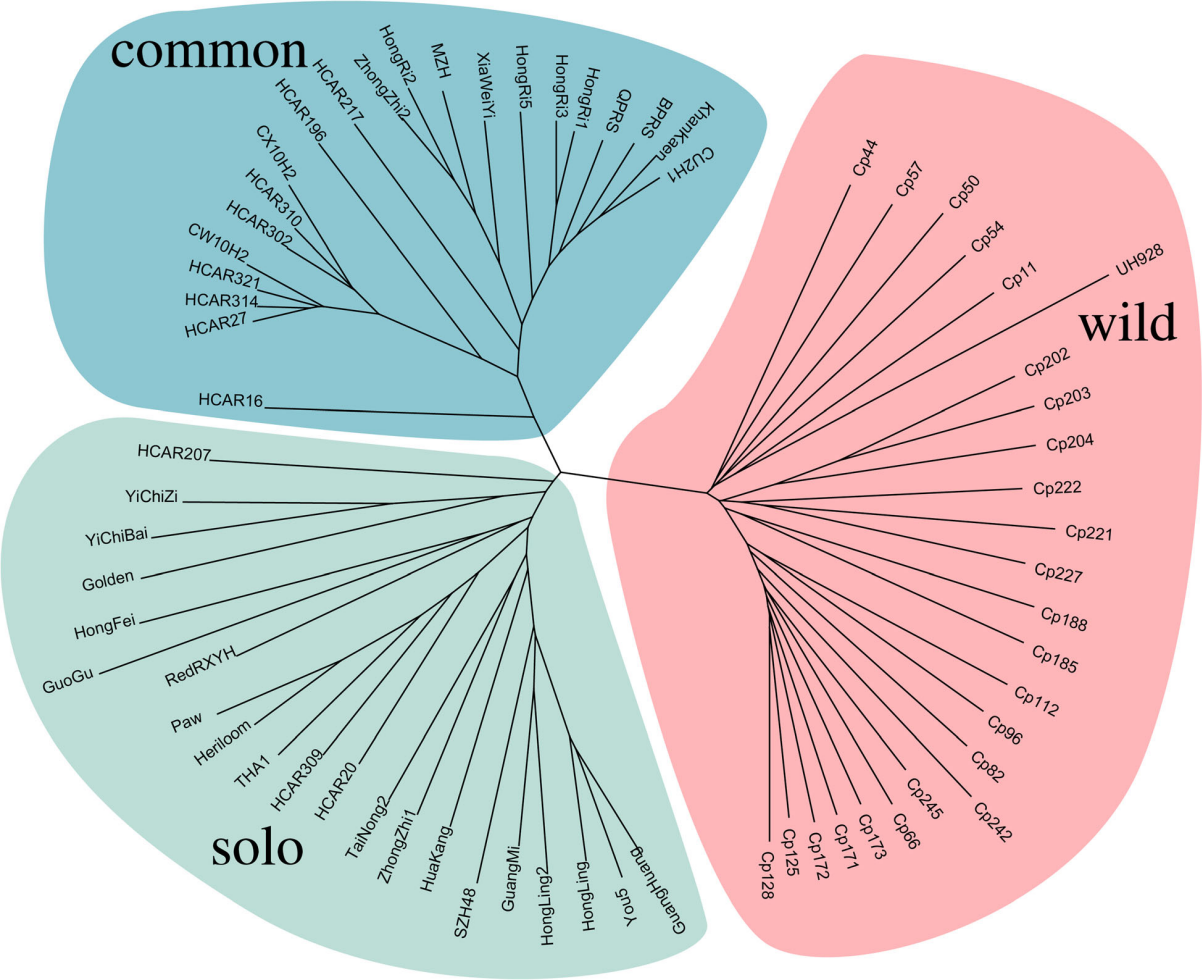
Neighbor-Joining phylogenetic tree based on filtered SVs

Phylogenetic analysis based on SVs distinguished four papaya groups, which were wild, solo and common showed that the papaya population was divided into 3 clusters. Costa Rica’s 25 wild populations were Wild branches. Twenty-one varieties, “solo”-type papaya, were divided into solo groups. And the other 21 varieties were common groups (Fig. 4).

We established different sets of structural variant genes according to the classification subgroups. By comparing the genes of cultivated and wild populations, we found some genes related to reproductive development, including gametophyte development, pollen tube growth, embryogenesis, embryo development, flowering time, peduncle growth, and multi-branched inflorescence formation (Table 1), of which the length of the pedicel is a unique phenotype in papaya evolution. We also detected some environmental adaptability related-genes which were related to heat stress, oxidative stress, salt stress, and defense responses (Table 1).

**Table 1:** Structural variant genes related to sexual reproduction and environmental adaptability

Gene name	Arabidopsis homolog	Gene Symbol	Subpopulation	Description
sunup.01G0016040	AT5G16560	KAN1	solo, common	the outer integument of the ovule, embryogenesis
sunup.07G0005810	AT1G06220	GFA1	solo, common	female gametophyte development, female fertility
sunup.07G0006190	AT1G29300	UNE1	solo	defects in pollen tube attraction
sunup.07G0010620	AT1G69940	PPME1	common	pollen tube elongation
sunup.03G0008270	AT1G04880	HMGBD15	common	pollen tube growth
sunup.01G0016020	AT2G26490	REN4	solo, common	negative regulator of pollen germination
sunup.06G0003180	AT1G50030	TOR	solo, common	embryogenesis, water-use efficiency and yield
sunup.06G0007900	AT1G09730	SPF1	solo	embryo development, fertility
sunup.06G0009160	AT4G38430	ROPGEF1	solo	embryos development
sunup.03G0008500	AT4G13750	EMB2597	common	embryo-defective
sunup.01G0026320	AT3G57230	AGL16	solo	flowering time
sunup.01G0008730	AT3G28860	PGP19/MDR1	solo	peduncle growth, multi-branched inflorescence
sunup.05G0008680	AT2G46240	BAG6	common	heat stress, fungal resistance
sunup.07G0000100	AT4G21320	HSA32	common	heat stress associated 32-kD protein
sunup.08G0015580	AT3G53990	USP	solo, common	heat shock and oxidative stress
sunup.03G0007120	AT2G05590	OCR2	solo	oxidative stress
sunup.06G0013090	AT4G38160	MTERF6	solo, common	defective chloroplasts or photosynthesis rate
sunup.01G0002550	AT1G65930	CICDH	solo	defense responses, pathogen responses
sunup.07G0001840	AT4G22330	ATCES1	solo, common	disease resistance, salt stress

### Analysis of artificially selected copy number variation genes

We identified 2526 and 3471copy number variant genes in common and solo cultivated populations, respectively. The two cultivated populations have similar distributions of copy number variant genes, which are concentrated in the middle and both ends of chromosomes like other structural variants, such as chr1, chr2, chr4, chr8, and chr9. However, the copy number-enriched region corresponds to the low structural variation region in chromosome 5 (Fig. 1).

Artificially selected CNVs can be identified by comparing of relative allele frequency (RAF) between wild and cultivated populations [8]. A total of 55 and 56 CNVs were under artificial selection in the solo and common subpopulations (Supplemental Table S3). These CNVs regions covers from 108 to 132 kb genomic regions and overlapped with 91 protein-coding genes, which were randomly distributed in different chromosomes in the two subgroups. Expression analysis found that 55 (60%) of these CNV-domesticated genes were expressed in different papaya tissues. There were highly expressed genes in various tissues (Supplemental Fig. S4). These expressed genes were enriched in six pathways [26] (Supplemental Table S4), namely peroxisome, glycosaminoglycan degradation, sesquiterpenoid and triterpenoid biosynthesis, cutin, suberine and wax biosynthesis, mapk signaling pathway and oxidative phosphorylation. These pathways are essential in the growth and development of plants.

In addition, the annotation of CNV-genes found that some genes related to reproduction were found in the solo and common subpopulations (Table 2), including *AGAMOUS-LIKE 80(AGL80), CELL DIVISION CYCLE 20.1(CDC20.1)* and *METHIONINE SYNTHASE 2(MS2)*. We also found three genes related to environmental stress response, including heat stress related gene *HSFA2*, drought-induced gene *RDUF2*, and *ASMT* gene encoding a cytosolic N-acetylserotonin O-methyltransferase gene that was related with salt stress. Four CNV-genes related to pathogen resistance were also found, *GDSL LIPASE 1(GLIP1), PATHOGENESIS-RELATED GENE 1 (PR1)* and two PR peptides belonging to the PR-6 proteinase inhibitor family.

**Table 2:** CNV-genes related to reproductive and environmental adaptability in different cultivated subpopulations

Gene name	Arabidopsis homolog	Gene Symbol	Subpopulation	Description
sunup.02G0014350	AT5G48670	AGL80	solo	central cell and endosperm development
sunup.01G0011520	AT4G33270	CDC20.1	solo	indispensable for meiosis and male fertility
sunup.08G0020230	AT3G11980	MS2	solo, common	male sterility
sunup.03G0024240	AT4G35160	ASMT	common	enhanced high light stress tolerance and salt tolerance
sunup.07G0012360	AT2G26150	HSFA2	solo, common	heat Tolerance
sunup.01G0012670	AT5G59550	RDUF2	solo, common	tolerance to drought stress
sunup.04G0018230	AT5G40990	GLIP1	solo, common	pathogen resistance
sunup.06G0008620	AT2G14610	PR1	solo	response pathogens
sunup.08G0014820	AT2G38870	AT2G38870	common	PR (pathogenesis-related) peptide
sunup.08G0014690	AT5G43570	AT5G43570	common	PR (pathogenesis-related) peptide

### Validation of structural variations

**Fig. 5:**
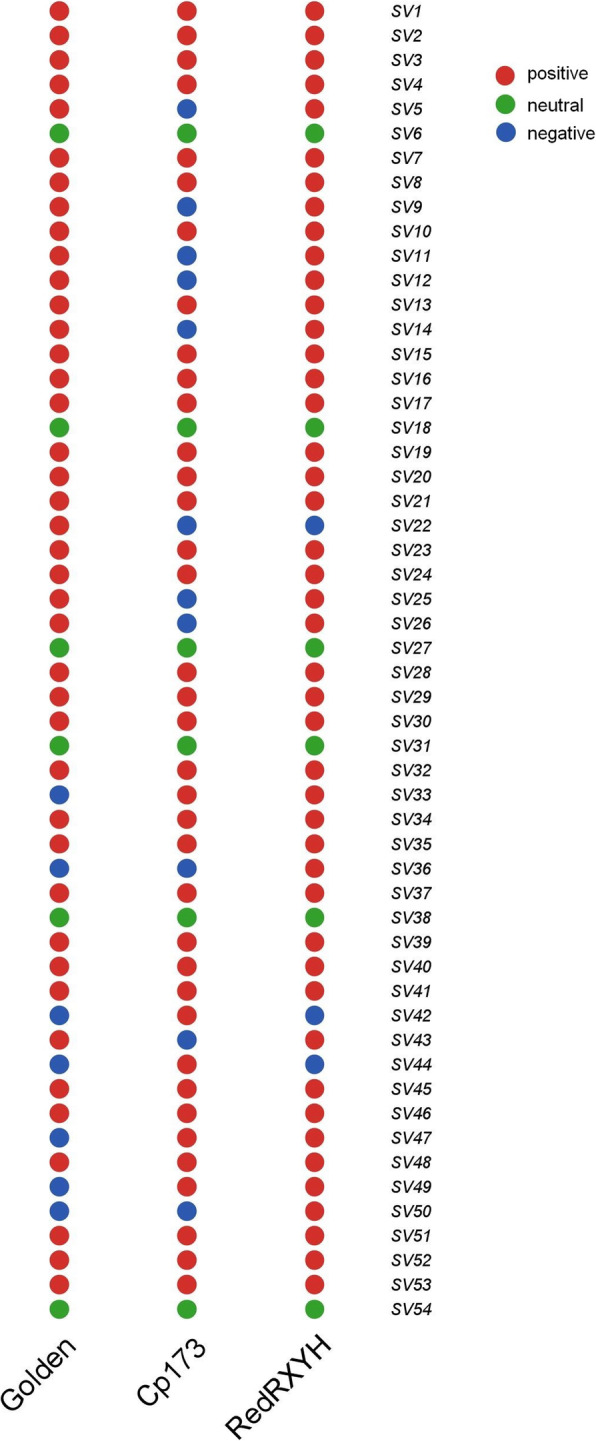
Statistical PCR amplification is consistent with predicted structural variations. Positive, consistent; negative, inconsistent; neutral, unable to amplify

We randomly selected 54 SVs loci to design primers to calculate the false discovery rate (FDR) of structural variations, including 22 insertions and 32 deletions (Supplemental Table S5). The total DNA of varieties Golden, Cp173 and RedRXYH were extracted and used to verify the primers. The reported autosomal universal primer 71E was used as a positive control in this paper^18^. We define PCR amplification that is consistent with the predicted SVs as positive, inconsistent as negative, and that cannot be amplified in three breeds as neutral. Six neutral primers were removed in the next statistical analysis (Supplemental Fig. S5). There were 123 positive and 21 negative amplitudes, with an FDR of 14.58%. PCR verification results show that the SVs has a high accuracy in this study (Fig. 5).

## Discussion

A large genome structural variant data set was generated in wild and cultivated papaya. A total of 8083 structural variants were detected. These SVs are unevenly distributed in papaya, mainly concentrated at the telomeric and centromeric regions of chromosomes (Fig. 1), which may be caused by genetic recombinations in telomeric regions and retrotransposon-mediated rearrangements in centromeric regions. Recent results also support the conclusion that large SVs are not evenly distributed on chromosomes [24]. However, the number of SVs (from 876 to 983) in each chromosome (except for chr8) is almost the same, suggesting that each chromosome may have been subjected to almost the same selection pressure in natural selection process. Most of the SVs is deletion type, indicating that deletions are very common during the evolution of the genome.

Through genome re-sequencing, it is possible to discover a large number of SNPs, Indels, SVs, and CNVs that can be used to develop molecular markers and provide genetic resources for molecular marker assisted breeding. The development of molecular markers in papaya is based on the SNP and InDel information, such as the molecular marker PMSM1 and PMSM2 for papaya sex identification [18]. However, large structural variations have not been thoroughly studied in papaya. This is the first time that structural variation has been detected in wild and cultivated populations of papaya and our results provide resources for the development of papaya molecular markers.

We identified some SV related genes and CNV related genes that may be artificially selected during the domestication and improvement of papaya. These candidate genes are very important in the sexual reproduction and agronomic traits of papaya, such as pistil development, female gamete development, pollen tube growth, embryo development, flower time, crop yields, and peduncle elongation. For example, *TOR* is involved in embryogenesis and is expressed in the embryo, endosperm, and meristem under Rapamycin [27, 28]. Interestingly, heterologous expression analysis revealed that *TOR* regulates genes in different developmental stages, as well as photosynthesis, productivity-related functions, and water use efficiency in rice [29]. *TOR* improves nutrient uptake efficiency and crop yields through autophagy-related methods [30]. *PGP19* has an ability to transport auxin through the cell membrane, and *pgp19* mutation can lead to abnormal distribution of auxin in the inflorescence stem in Arabidopsis [31]. PGP19 interacts with auxin efflux carrier proteins PINs (PIN1 and PIN2) to regulate the distribution of auxin in plants, thereby affecting the elongation of inflorescence stem [32]. The discovery of these genes provides an opportunity to better understand the domestication of hermaphrodite papaya from male plants about 4000 years ago [20].

The evolution of plants is a process of continuous adaptation to various and changing environments. We have also detected some selected structural variant genes and/or CNV-genes related to environmental adaptability. *ASMT* is a terminal enzyme for melatonin synthesis, and *ASMT* gene expression is up-regulated under strong light and salt stress, which leads to an increase in melatonin and thus improving salt tolerance [33]. Moreover, melatonin is involved in the tolerance of plants under high light stress [34]. The up-regulation of *GLIP1* in plants enhances resistance to pathogens, including *Pseudomonas aeruginosa*, *Erwinia carotovora* and *Pseudomonas syringae*. In addition, local treatment with GLIP1 protein can activate plant systemic resistance, inducing resistance gene expression and pathogen resistance in leaves [35]. These candidate genes might play important roles in environmental adaptability during domestication of papaya.

## Conclusions

Structural variations exist widely in plant genomes and plays an important role in evolution, growth, and development. Here, we use population genomic data to search for papaya genome-wide structural variations. We detected a total of 8083 structural variants in the whole genome, including 5260 deletions, 552 duplications, and 2271 insertions. A total of 1794 coding proteins were detected in the SV region, of which 75% (1350) of the genes were expressed in at least one tissue. Gene function analysis found that SV-genes are involved in the growth and development of papaya. Further WGCNA analysis revealed the co-expression relationship between SV-genes and different tissue samples, and functional enrichment analysis of different module genes revealed that it was mainly related to biological growth and environmental response. We also identified some SVs and CNVs genes that were artificially selected during the domestication of papaya. These genes are related to environmental adaptability, sexual reproduction, and important traits, such as pistil development, embryonic development, flowering time, crop yield, pedicel elongation, defense response, and pathogen response.

## Materials and methods

### Sample collection and whole-genome re-sequencing

We collected 35 samples of Illumina reads from the NCBI BioProject database (http: //www.ncbi.nlm.nih.gov/bioproject) under accession number PRJNA271489 for this project research (Supplemental Table S1) [20]. 31 hermaphrodite and 1 male papaya plants were collected from China, and fresh leaf tissue were dried on silica gel in the field and stored at 80 °C.

Genomic DNA was extracted from leaf tissues using Plant DNA Isolation Reagent kit (TaKaRa). Plant genomic DNA was fragmented with about 500 bp size fragment using an ultrasonic disruptor. Paired-end DNA libraries with an average insert size of 500 bp were built using the Illumina DNA library kit according to the manufacturer’s instructions and sequenced on an Illumina Hiseq 2500 at 150 bp length.

### Read alignment and SV calling

For the re-sequenced genome data, FastQC was used to check the quality of raw sequencing data [36]. Raw reads were filtered to remove low quality bases and trimmed for indexes using Trimmomatic (v.0.33) prior to alignment [37]. Illumina sequence adaptors and other Illumina-specific sequences from the read were removed, the start or end bases of a read were trimmed when below a threshold quality (below quality 3) and N (undetermined) base pairs also were removed, and reads were scanned using a 4 bp sliding window and trimmed when the average quality per base dropped below 30, and specifies the minimum length (100 bp) of reads to be kept, yielding clean data for further analysis. Clean reads were aligned to the new papaya genome sequence. The Burrows–Wheeler Aligner was used for read alignment using strict parameters, and we use the faster and more accurate BWA-MEM algorithm. SAMtools was used to convert the obtained SAM file to a BAM file and remove duplicate reads [38]. SVs were called from mapped paired-end sequencing reads using the Manta pipelines [39].

### Gene expression analysis

The ovule, pollen and stamen tissues were collected in the greenhouse, and RNA was extracted. Each RNA-seq library was constructed separately using NEBNext Ultra RNA Library Prep Kit for Illumina following the protocol. A total of nine cDNA libraries were sequenced on the Illumina HiSeq2500 platform to generate a larger number of paired-end sequence reads. Raw RNA sequence data for each library is publicly available on NCBI BioProject, under the accession number: PRJNA693693 (https://www.ncbi.nlm.nih.gov/bioproject/PRJNA693693). Besides these libraries, RNA sequencing data from pistil (Accession number: PRJNA532376), normal leaf (Accession number: PRJNA555541), and flower (Accession number: PRJNA555541), were downloaded from the Sequence Read Archive (SRA) database.

The low quality bases were removed as done in DNA re-sequencing analysis. The clean reads were aligned to the papaya genome sequences by Bowtie2 with the end-to-end model default parameter, and each gene FPKM (Fragments Per Kilo-base pair per Million reads) value was calculated using RSEM [40].

### The weighted correlation network analysis (WGCNA)

WGCNA software package includes functions such as weighted co-expression network construction, gene module testing, screening hub genes, computing topological characteristics, and mapping [41]. WGCNA is used to calculate the pairwise correlation coefficient between genes to obtain the similarity matrix, and select the soft threshold according to the functions of pickSoftThreshold. The soft threshold is a criterion based on the approximate scale-free network to make the constructed network conform to the power law distribution, and this method strengthens the strong correlation and weakens the weak correlation, making the correlation value more consistent with the scale-free network characteristics and more biologically significance. In this study, when the soft threshold is 16, the gene topology matrix expression network is closest to the scale-free distribution, and the high connectivity can be guaranteed. Through further analysis of modules, the differences between modules are calculated and a tree of modules is constructed. The minimum number of genes in the module is set to 30, and the threshold of the cutting height is set to 0.2 to merge modules with higher similarity. The module feature vector was used to find the module most related to the phenotype, the correlation coefficient between the feature vector gene and the phenotype was calculated by cor functions, and the *P* value was calculated by functions corPvalueStudent. The modules with the highest correlation coefficient and the lowest *P* value were selected for further functional analysis. Genes of the same gene module may exist in the same metabolic pathway or have similar functions.

### GO and KEGG enrichment analysis

The clusterProfiler package of R software was used for functional analysis of trait-related module genes [42]. The GO and KEGG enrichment analysis were performed using the enrichGO and enrichKEGG functions, respectively. The barplot and cnetplot functions were used to draw bubble chart and histogram.

### Population genetic analyses

Our analysis of Illumina population data called SVs, including INS, DEL and DUP. Generally, variation calls for short-read alignments allow us to detect variants without breakpoint using Manta, and some SVs has multiple breakpoints in the population. Finally, we only retained SVs that shared the same and clear breakpoints across the population samples for phylogenetic analyses. VCF2Dis and the Neighbor-Joining with 1000 boostrap methods were used to convert the structure-variant Variant Call Format file into a pdistance matrix. PHYLIPsoftware was used to construct a phylogenetic tree of papaya (https://github.com/BGI-shenzhen/VCF2Dis) [43]. We use MEGA X to view the phylogenetic tree and add the name of the population [44].

## Supplementary Information

**Additional file 1: Supplemental Figure S1.** Bubble chart of GO enrichment for genes with overlapping SV and CDS regions.

**Additional file 2: Supplemental Figure S2.** KEGG enrichment histogram of genes with overlapping SV and CDS regions.

**Additional file 3: Supplemental Figure S3.** WGCNA module aggregation diagram

**Additional file 4: Supplemental Figure S4.** Heat map of the expression of 91 CNV-genes in different tissues of papaya.

**Additional file 5: Supplemental Figure S5.** Amplification of SVs fragments using different SVs markers in three breeds. Each primer was amplified in these three varieties, Golden, Cp173, and RedRXYH. 71E was used as a positive control that amplifies an autosomal fragment from all papaya types. M 2000 bp DNA marker ladder.

**Additional file 6: Supplemental Table S1.** The re-sequencing samples used in this study.

**Additional file 7: Supplemental Table S2.** Statistics of structural variation of different chromosomes in papaya.

**Additional file 8: Supplemental Table S3.** Statistics of the number of chromosome CNV-genes in different cultivated subgroups.

**Additional file 9: Supplemental Table S4.** 91 CNV-genes function enrichment meaningful pathwayc

**Additional file 10: Supplemental Table S5.** PCR primers information used in this paper

## Data Availability

The re-sequencing datasets used and analyzed during the current study are publicly available on NCBI BioProject, under the accession number: PRJNA693144 (https://www.ncbi.nlm.nih.gov/bioproject/PRJNA693144). The RNA-seq raw datasets were uploaded to NCBI BioProject, under the accession number: PRJNA693693 (https://www.ncbi.nlm.nih.gov/bioproject/PRJNA693693).
