# Supplementary figures and images for: Structural variations in papaya genomes
Source: BMC Genomics. 2021 May 10;22:335. doi: 10.1186/s12864-021-07665-4 (PMC8108470; doi:10.1186/s12864-021-07665-4)

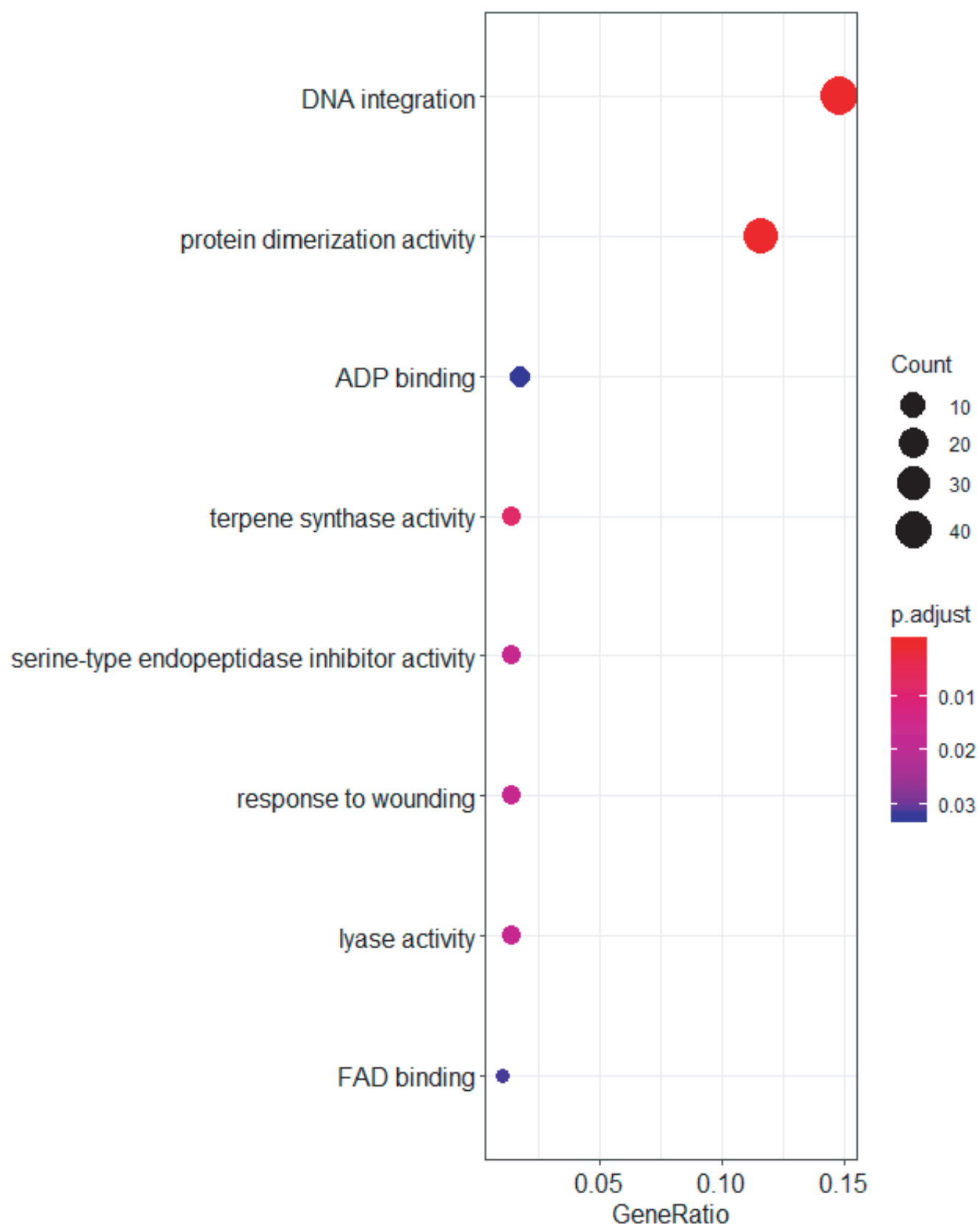

Supplement: Supplementary file 1 — Additional file 1: Supplemental Figure S1. Bubble chart of GO enrichment for genes with overlapping SV and CDS regions. [file 12864_2021_7665_MOESM1_ESM.pdf]

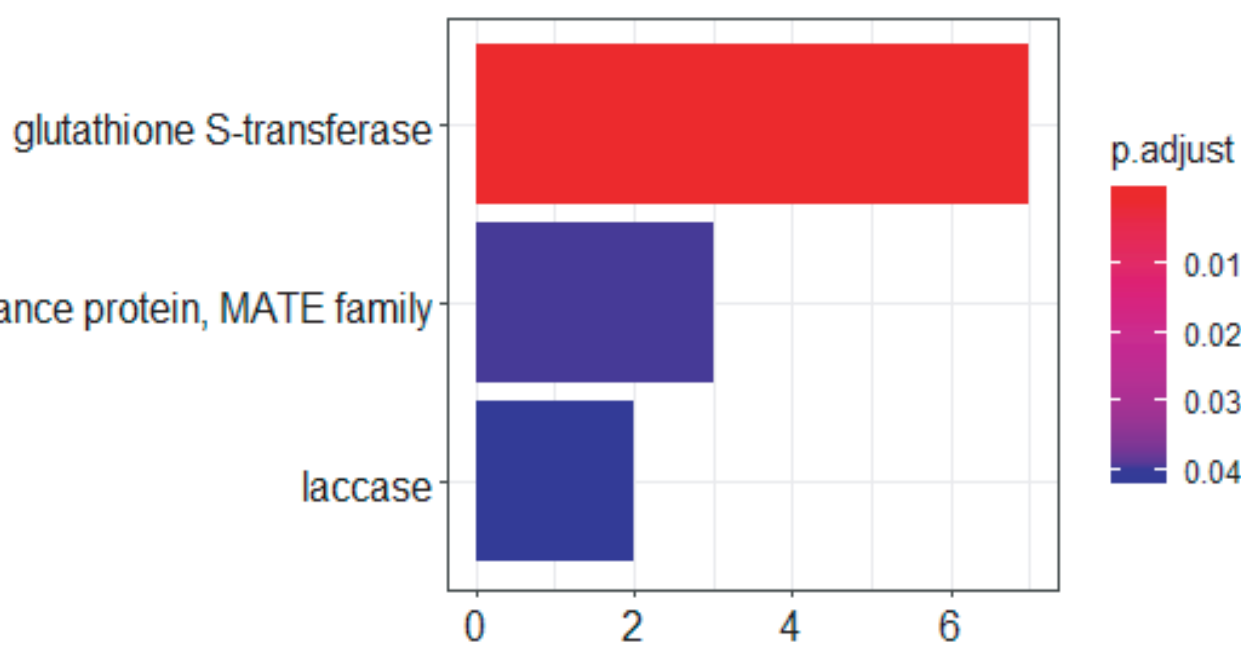

Supplement: Supplementary file 2 — Additional file 2: Supplemental Figure S2. KEGG enrichment histogram of genes with overlapping SV and CDS regions. [file 12864_2021_7665_MOESM2_ESM.pdf]

Cluster Dendrogram

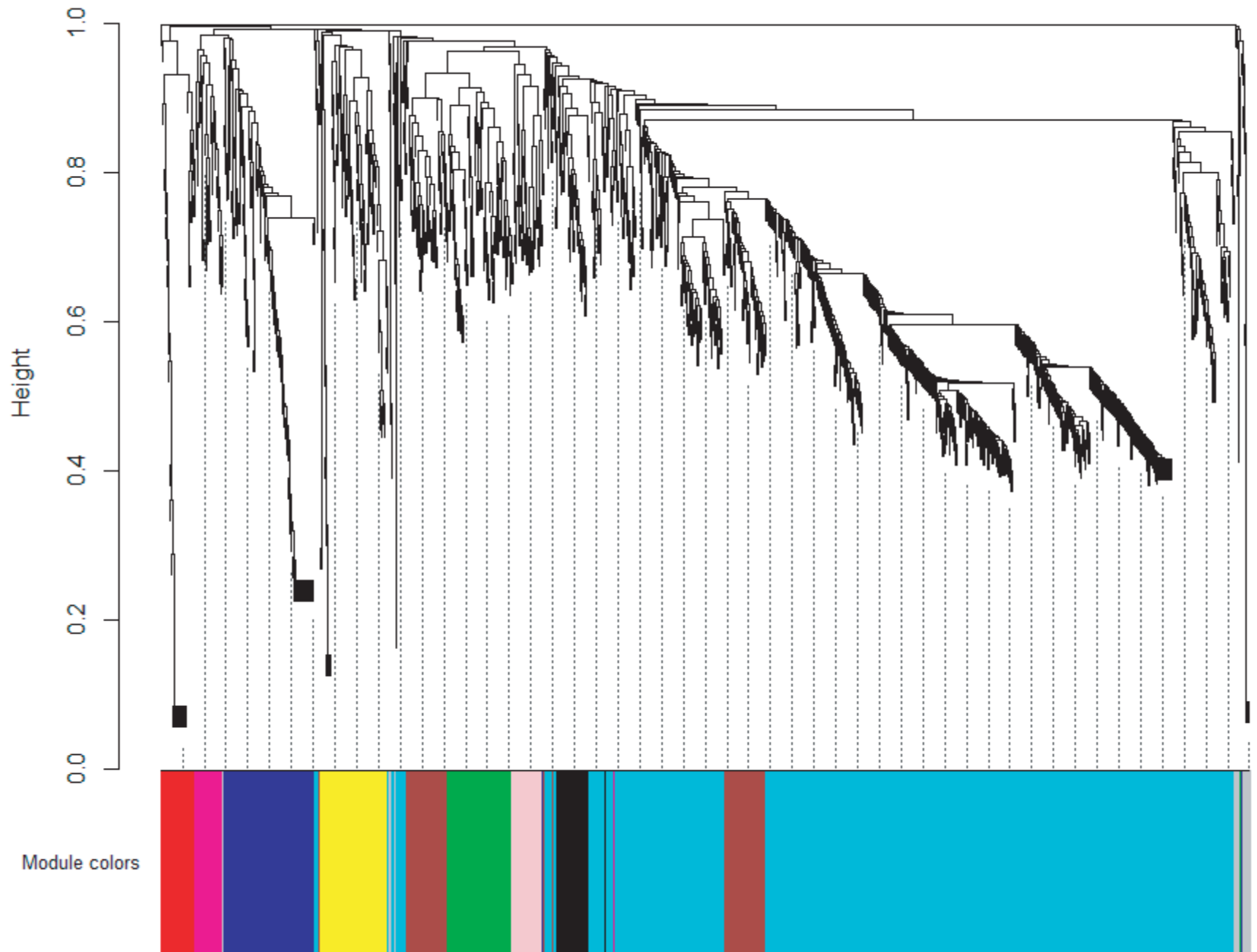

Supplement: Supplementary file 3 — Additional file 3: Supplemental Figure S3. WGCNA module aggregation diagram [file 12864_2021_7665_MOESM3_ESM.pdf]

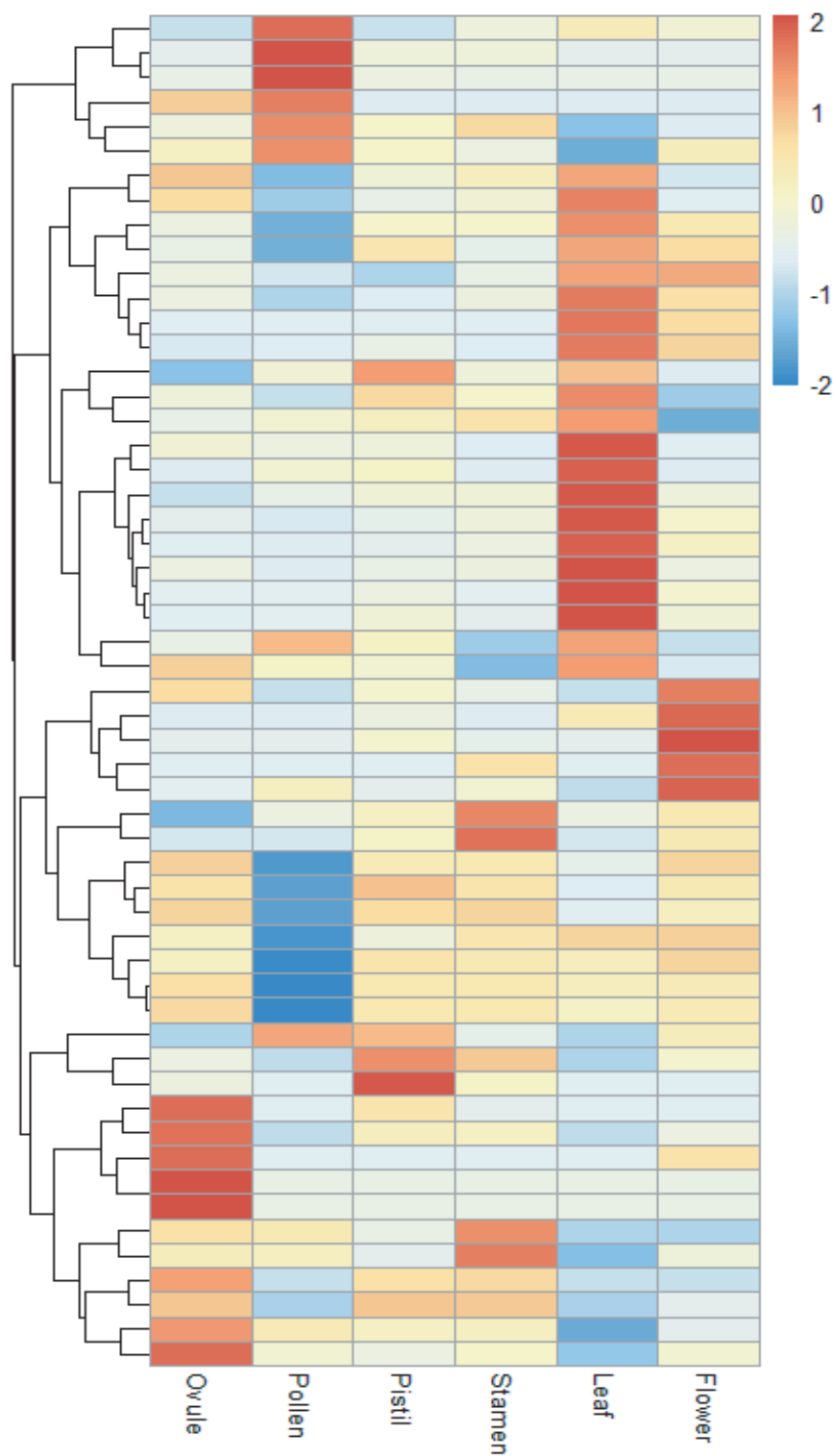

Supplement: Supplementary file 4 — Additional file 4: Supplemental Figure S4. Heat map of the expression of 91 CNV-genes in different tissues of papaya. [file 12864_2021_7665_MOESM4_ESM.pdf]

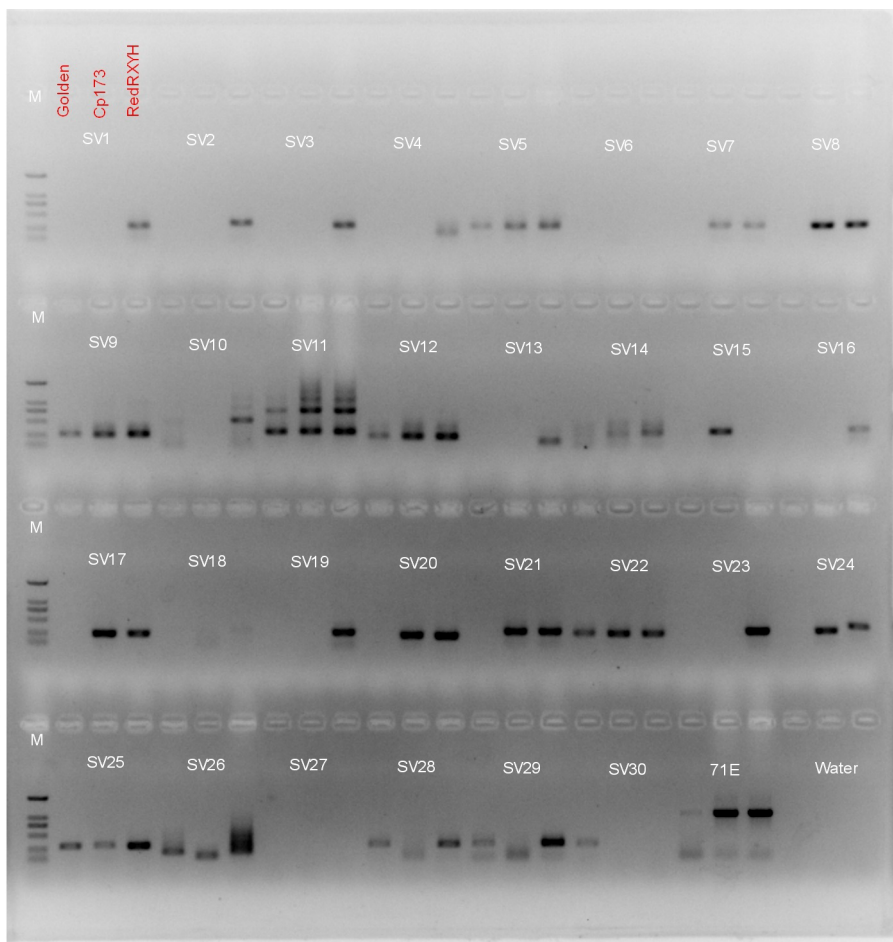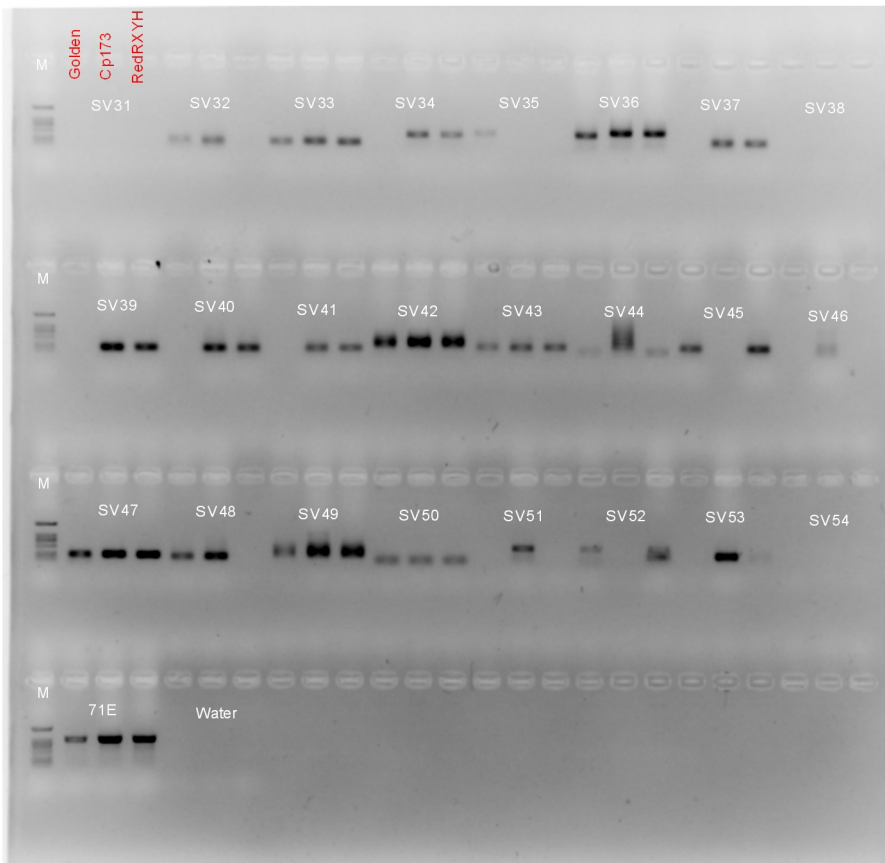

Supplement: Supplementary file 5 — Additional file 5: Supplemental Figure S5. Amplification of SVs fragments using different SVs markers in three breeds. Each primer was amplified in these three varieties, Golden, Cp173, and RedRXYH. 71E was used as a positive control that amplifies an autosomal fragment from all papaya types. M 2000 bp DNA marker ladder. [file 12864_2021_7665_MOESM5_ESM.pdf]
